# The Popliteofibular Ligament: A Narrative Review of Anatomical Variants and Surgical Relevance

**DOI:** 10.3390/jcm14165667

**Published:** 2025-08-11

**Authors:** Łukasz Olewnik, Ingrid C. Landfald, Bartosz Gonera, Łukasz Gołek, Kacper Ruzik, Robert F. LaPrade

**Affiliations:** 1 Department of Clinical Anatomy, Mazovian Academy in Płock, 09-402 Płock, Poland; ingridceciliee@gmail.com (I.C.L.); b.gonera@mazowiecka.edu.pl (B.G.); l.golek@mazowiecka.edu.pl (Ł.G.); k.ruzik@mazowiecka.edu.pl (K.R.); 2 VARIANTIS Research Laboratory, Department of Clinical Anatomy, Mazovian Academy in Płock, 09-402 Płock, Poland; 3 Twin Cities Orthopedics, Edina, MN 55435, USA; laprademdphd@gmail.com

**Keywords:** popliteofibular ligament, knee stability, anatomical variations, posterolateral corner, surgical anatomy, clinical implications

## Abstract

The popliteofibular ligament (PFL) plays a vital role in knee joint stability, particularly within the posterolateral corner (PLC) of the knee. Located between the femoral condyle and the fibular head, the PFL resists excessive external rotation and lateral translation of the tibia, thus preventing knee instability during dynamic activities. This ligament, although integral in maintaining knee integrity, has often been overlooked in clinical practice and research. This review synthesizes the current literature on the anatomy, biomechanics, and clinical relevance of the PFL, highlighting its morphological variations, functional significance, and implications for knee injuries, particularly in relation to PLC trauma. Anatomical studies have identified significant variations in the PFL’s structure, including single, bifurcated, and double ligament forms, each influencing the ligament’s mechanical properties and its susceptibility to injury. Additionally, the PFL’s interaction with other knee structures, such as the fibular collateral ligament and popliteus tendon, is crucial for resisting rotational and translational forces, especially during high-stress movements like pivoting and cutting. Injuries to the PFL, often occurring in conjunction with other PLC structures, can lead to chronic knee instability and require precise diagnostic techniques, including MRI and ultrasound, for accurate assessment. Surgical management, including PFL reconstruction, has shown promising results in restoring knee stability, especially when tailored to the patient’s anatomical variant. This review provides a comprehensive understanding of the PFL’s role in knee function and its clinical implications, emphasizing the need for individualized treatment strategies in knee reconstruction

## 1. Introduction

The popliteofibular ligament (PFL) is a key stabilizing structure within the posterolateral corner (PLC) of the knee. Connecting the popliteus tendon (PT) to the fibular head, the PFL plays a critical role in resisting external tibial rotation and lateral translation, thereby maintaining joint stability during dynamic and weight-bearing activities [1,2]. Despite its biomechanical importance, the PFL has historically been underrepresented in clinical and anatomical literature compared to other structures such as the anterior and posterior cruciate ligaments [3].

Recent anatomical and biomechanical investigations have enhanced understanding of the PFL’s function, morphology, and clinical relevance [4,5,6]. Notably, variations in its structure including single-band, bifurcated, and double-band types may influence both the risk of injury and surgical outcomes [7]. The PFL functions synergistically with the posterior cruciate ligament (PCL), fibular collateral ligament (FCL), and PT, forming a complex stabilizing network that is essential during rotational movements such as pivoting or cutting [8,9]. Injuries to the PFL, often occurring in conjunction with other PLC structures, can result in chronic knee instability if left undiagnosed or improperly treated [10].

The present review aims to synthesize anatomical, biomechanical, and clinical knowledge of the PFL to support diagnosis, treatment, and future research related to posterolateral knee injuries. Particular emphasis is placed on the morphological variability of the PFL, its interactions with adjacent structures, comparative anatomy across species, and its implications for surgical reconstruction. Additionally, the review highlights existing knowledge gaps, including the absence of standardized classification systems and limited data on anatomical differences related to sex, age, and ethnicity.

## 2. Methods

A literature review was conducted to identify relevant studies describing the anatomy, morphological variability, and clinical significance of the PFL. The following databases were searched: PubMed, Scopus, and Web of Science, covering articles published up to April 2025. The search was performed using a combination of the following keywords: “popliteofibular ligament”, “posterolateral corner”, “PFL anatomy”, “knee stability”, “PLC reconstruction”, “ligament morphology”, and “knee biomechanics”.

Only peer-reviewed articles written in English were included. The inclusion criteria encompassed original anatomical, radiological, and clinical studies as well as relevant systematic and narrative reviews that provided detailed information on the PFL. Exclusion criteria were studies without direct reference to the PFL, case reports with insufficient anatomical description, and non-peer-reviewed literature.

Titles and abstracts were screened independently by two authors. The full texts of selected articles were reviewed for relevance, and references within these articles were also scanned for additional sources.

## 3. Anatomy of the Popliteofibular Ligament

The PFL is a fundamental stabilizer within the PLC of the knee, counteracting excessive external tibial rotation and lateral translation during dynamic, weight-bearing activities. Despite being less extensively studied than the anterior posteriori cruciate ligament (ACL) and PCL, the PFL is increasingly recognized for its critical biomechanical and clinical relevance [2,3].

Anatomically, the PFL originates from the PT, not from the femoral condyle, and extends obliquely to the posterior aspect of the fibular head. Positioned posterior to the FCL, it forms part of a complex network with the FCL, biceps femoris tendon, and other posterolateral stabilizers [1,11]. Given its structural variability and deep location, precise anatomical knowledge is vital for both diagnosis and reconstructive strategies [4,5].

### 3.1. Location and Course

The PFL runs from the popliteus musculotendinous junction obliquely across the posterolateral knee to the fibular head. Its trajectory lies posterior to the FCL and PT, inserting just below the biceps femoris attachment [3,7,12]. This orientation allows the PFL to effectively resist varus forces and external rotation during motion [1,11].

### 3.2. Structure and Histology

Histologically, the PFL is composed of densely packed Type I collagen fibers arranged in a crimped pattern for tensile strength and controlled stretch under load [4]. A smaller proportion of elastic fibers contributes to flexibility and recoil [5]. Fibrocartilage at the femoral and fibular attachments provides shock absorption and protects against stress-related degeneration [9,13].

### 3.3. Size, Shape, and Individual Variability

The PFL typically measures 10–14 mm in length (mean approximately 11.8 mm) and 8–10 mm in width at its midpoint, with these dimensions varying according to sex, age, and physical activity [2,3]. Generally, males and younger individuals possess larger and thicker ligaments, whereas aging is associated with ligamentous atrophy, thinning, decreased elasticity, and diminished function, potentially compromising joint stability [4,5,14].

Morphologically, the PFL may present in various shapes such as triangular, rectangular, or fan-shaped. Common anatomical variants include single-band and bifurcated (dual-band) ligaments, with occasional broader origins or multiple insertions. The insertion sites of the PFL also vary substantially; although the ligament is most commonly attached at the apex of the fibular head, insertions can occur at different regions of the fibular styloid process, which may influence the ligament’s stabilizing role during varus or rotational stress [4,5]. In some cases, bifurcations are present, forming two distinct bands that anchor separately to the fibula, potentially offering increased resistance to lateral tibial translation [15].

Demographic differences have also been reported. For example, East Asian populations tend to exhibit smaller and thinner PFLs with different insertion patterns compared to Caucasians, which may influence susceptibility to posterolateral instability [3,16]. Differences between sexes are thought to be related to variations in muscle mass and joint loading, with males tending to have longer and thicker PFLs than females [15,17].

These structural and demographic variations are clinically significant, especially in the context of knee instability and PL injuries. The anatomical variability in size, shape, and structural configuration of the PFL significantly influences its biomechanical properties and functional role in knee stability. Recognition of these variations is crucial for accurate diagnostic imaging interpretation and for tailoring surgical reconstruction techniques to individual patient anatomy, ultimately improving clinical outcomes and reducing the risk of residual instability. Understanding the diverse anatomical presentations of the PFL can help clinicians tailor diagnostic, surgical, and rehabilitative strategies more effectively.

## 4. Morphological Variations in the Popliteofibular Ligament

The PFL demonstrates significant morphological variability, particularly in its size, shape, and the arrangement of its fibers. These variations can impact the ligament’s function and its role in knee stability, especially in the context of dynamic movements and knee joint pathology. The PFL can vary in terms of its attachment points, length, and the presence of accessory bands or bifurcated structures.

In the most common form (Type I), the PFL consists of a single ligament that attaches to the apex of the fibular head. However, some individuals exhibit a bifurcated PFL (Type II), where the ligament divides into two bands, with one inserting onto the anterior slope and the other onto the posterior surface of the fibula. A rarer variation, Type III, involves a double PFL, where two distinct bands of the ligament originate from different parts of the PT and musculotendinous junction, with separate insertions on the fibular styloid process [2,4].

These variations in the PFL are clinically significant, as they can influence the biomechanical properties of the knee and its response to injury or surgery. The presence of accessory bands or a double PFL may offer enhanced stability to the knee, potentially affecting both its normal function and its vulnerability to injury. As such, understanding these morphological differences is important for clinicians, particularly when evaluating knee instability or planning reconstructive surgery [14].

Comparative Note

Compared to other key components of the PLC such as the FCL, the arcuate ligament complex, and the lateral gastrocnemius tendon, the PFL exhibits a substantially higher degree of morphological variability. While structures like the FCL and arcuate complex typically present with consistent anatomical patterns and attachment sites, the PFL is marked by a heterogeneous spectrum of types and insertions. This diversity carries important implications for imaging interpretation, surgical planning, and individualized clinical decision-making.

### 4.1. Classification System

**Figure 1:**
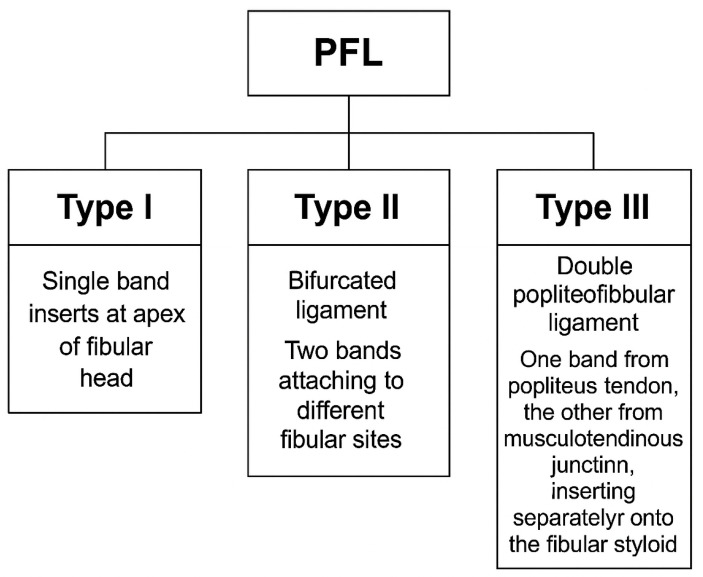
Classification of the popliteofibular ligament (PFL) into Type I, Type II, and Type III, based on Olewnik et al. (2022) [7].

**Figure 2:**
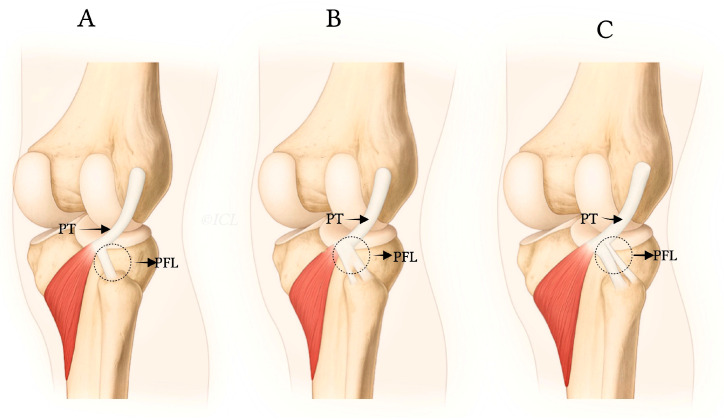
Anatomical illustrations showing distal attachments and morphologies of PFL types I (**A**), II (**B**), and III (**C**), as described by Olewnik et al. [7].

The PFL classification proposed by Olewnik et al. [7] categorizes the ligament into three primary types based on its distal attachment and structural characteristics (see Table 1 and Figure 1 and Figure 2):

- **Type I:** Single-band ligament attaching to the apex of the fibular head, representing the most common morphology (72.3%). This type is generally considered the most biomechanically stable variant and serves as the standard reference in clinical practice.
- **Type II:** Bifurcated ligament consisting of two distinct bands attaching to different surfaces of the fibula (8.7%). This anatomical complexity may enhance stability but also increases surgical complexity.
- **Type III:** Double PFL with two separate ligaments originating from the PT and the musculotendinous junction, inserting at distinct fibular styloid surfaces (7.3%). This variant provides potentially superior native stability but presents challenges in accurate surgical reconstruction due to its spatial complexity [7,12].

The classification details are summarized in Table 1, which provides the prevalence and descriptions of each type. Furthermore, Figure 1 and Figure 2 visually depict these types, illustrating the morphological differences and their anatomical insertions.

Clinically, awareness of these types is critical. The presence of bifurcated or double-band variants (Types II and III) can complicate diagnostic imaging interpretation and surgical management. For example, the complex anatomy of Type III PFL may be difficult to fully visualize with standard MRI or US protocols, potentially leading to underdiagnosis or misinterpretation of ligament integrity [18,19].

From a surgical standpoint, reconstruction strategies must be tailored to the specific PFL type. While Type I variants may be adequately addressed with single fibular tunnel graft fixation, Types II and III require more precise graft placement, potentially involving multiple tunnels or advanced fixation techniques to restore native biomechanical function and avoid residual posterolateral instability [20,21].

Furthermore, knowledge of these variants aids in preoperative planning through detailed imaging assessment, allowing surgeons to anticipate anatomical challenges and optimize operative outcomes. Failure to recognize and reconstruct the PFL accurately, particularly in Types II and III, can result in persistent rotational laxity and graft failure [22].

Therefore, this classification system not only provides a standardized framework for anatomical description but also guides personalized clinical decision-making, improving both diagnostic accuracy and surgical success in managing posterolateral corner injuries.

### 4.2. Clinical Relevance of Variability

The morphological variability of the PFL, as classified in Table 1 and illustrated in Figure 1 and Figure 2 [7], has significant clinical implications. This variability may influence knee function, stability, and susceptibility to injuries or pathologies, particularly within the PLC of the knee. The PFL plays a crucial role in stabilizing the knee during dynamic movements such as pivoting and cutting. Variations in size, shape, and structural composition of the PFL can affect its ability to perform this stabilizing function effectively, potentially leading to increased knee instability or higher injury risk.

#### 4.2.1. Influence on Knee Function

The size and shape of the PFL, as well as its attachment sites, directly impact its biomechanical properties. Type I PFLs, consisting of a single ligament attaching to the apex of the fibular head, are generally larger and more robust, providing greater stability to the knee joint. Types II and III, characterized by bifurcated bands or double ligament structures, respectively, may confer enhanced stability due to additional supporting bands, thus offering potentially improved resistance to lateral tibial translation and external rotation during high-impact or rotational activities. However, these complex structures might also increase injury risk, especially if one of the bands is torn or damaged during intense physical activities involving rapid changes in direction [2,16].

#### 4.2.2. Increased Risk of Injury and Pathologies

Morphological variations can influence an individual’s susceptibility to knee injuries or pathologies. Smaller or thinner PFLs, often observed in females or older individuals, may be less capable of resisting external tibial rotation and lateral translation, thereby increasing the likelihood of PLC injuries and resultant knee instability. Such instability may lead to chronic conditions, including degenerative joint changes and osteoarthritis, if left untreated.

Conversely, larger, thicker PFLs, more common in males and younger individuals, typically provide better stability and may reduce injury risk. However, rarer types such as Type III, with two separate ligament bands, may complicate clinical management. For instance, the rupture of one band in a double PFL could be partially compensated by the remaining band, potentially delaying diagnosis and leading to more significant instability over time [2,14].

#### 4.2.3. Olewnik et al.’s Classification and Clinical Implications

The classification system proposed by Olewnik et al. [7], detailed in Table 1 and illustrated in Figure 1 and Figure 2, provides a framework to understand how different PFL types affect knee stability. Type I, the most common and stable variant, offers the greatest resistance to instability, while Types II and III with their complex morphologies may enhance stability but pose surgical and diagnostic challenges. Recognition of these types aids clinicians in predicting knee function and planning individualized treatment, including surgical reconstruction and rehabilitation protocols [7].

Additionally, the variability highlights the necessity of tailored clinical evaluation and imaging. Patients presenting with knee instability or PLC injuries should be assessed with attention to PFL morphology, as the presence of complex variants may require alternative surgical strategies compared to the more typical single-band ligament [14].

### 4.3. Surgical Implications of PFL Variants

#### 4.3.1. Clinical Considerations for Surgeons

A thorough understanding of PFL variants is essential for surgeons involved in PLC reconstruction. The anatomical diversity of the PFL, especially in its attachment sites, number of bands, and dimensional variability can significantly impact both surgical approach and clinical outcomes. Inadequate recognition of these variants may lead to incomplete stabilization of the PLC, persistent instability, or graft failure.

Recent studies emphasize the need to identify the type of PFL preoperatively using MRI or intraoperative exploration to adapt surgical planning accordingly [18,22]. Espinosa et al. [19] advocate for patient-specific planning in multiband PFL cases, suggesting that the standard fibular-based tunnel may be insufficient in complex configurations, particularly when the ligament displays bifurcation or dual origin.

Moreover, the use of allografts or autografts should be tailored to the anatomical variant present. In single-band PFLs (Type I), standard graft routing through a fibular tunnel may suffice. However, in Type II and especially Type III variants, graft fixation requires careful orientation and potentially multiple bone tunnels to mirror the native ligament architecture [20]. Misalignment or oversimplification of this complex anatomy risks insufficient rotational control, leading to residual varus or posterolateral rotatory instability [21].

Surgeons should also consider the biomechanical interaction between the PFL and adjacent structures such as the FCL, PT, and arcuate complex. Neglecting the multidirectional stabilizing role of the PFL, particularly in its more elaborate forms, can undermine the success of a global PLC reconstruction strategy.

#### 4.3.2. Surgical Caution for Type III Variant

Among the classified anatomical types, Type III PFL poses the greatest surgical challenge due to its double-banded configuration originating from two distinct sites: the PT and the musculotendinous junction. This variant has been shown to offer enhanced native knee stability but simultaneously complicates graft replication due to its spatial complexity and multi-anchor topology [18].

Espinosa et al. [19] highlight the increased surgical difficulty in Type III cases, emphasizing the need for dual graft pathways or innovative suture-bridge techniques to restore the native vector forces. The authors recommend intraoperative mapping of ligament orientation and selective use of imaging or dynamic fluoroscopy for tunnel placement, especially when fibular morphology or previous surgeries compromise the available bone stock.

Moreover, Type III may demand more extensive soft-tissue dissection and increased operative time. Poffenberger et al. [22] suggest considering alternative approaches such as gracilis tendon autografts with staged fixation in high-demand athletes or revision scenarios. These methods allow for more controlled graft tensioning and anatomic restoration in complex ligament configurations.

Senevirathna et al. [21] further warn that neglecting one of the two ligamentous components in Type III may paradoxically create iatrogenic instability, with the residual native band adapting maladaptively to altered mechanical loads. Hence, full replication of both bands, with anatomically accurate anchoring points, is crucial for successful surgical outcomes and long-term joint stability.

Mestriner et al. [20] conclude that Type III variants may require the surgeon to deviate from standard LaPrade-based PLC reconstructions, and propose a modified tunnel geometry protocol that considers both fibular and tibial vector forces. They also stress the importance of adapting surgical techniques to the biomechanical complexity of these reconstructions. Surgical teams must plan accordingly, incorporating preoperative imaging and intraoperative navigation tools to optimize outcomes.

## 5. Functions of the Popliteofibular Ligament

The PFL plays a vital role in stabilizing the knee, primarily by controlling external tibial rotation and preventing lateral translation. Situated within the PLC, it acts synergistically with the FCL, PT, and joint capsule to maintain knee integrity during dynamic and weight-bearing activities [1,23].

### 5.1. Knee Joint Stabilization

The PFL resists excessive external rotation of the tibia relative to the femur, a function critical during cutting, pivoting, and other rapid directional changes that pose a risk of knee instability [1,2]. By stabilizing the lateral femoral condyle and fibular head, it limits abnormal tibial rotation and protects key structures such as the ACL [1,2].

Additionally, the PFL helps prevent lateral tibial translation, especially when the knee experiences varus stresses during high-impact or lateral movements. Its cooperation with the FCL enhances resistance to these forces, ensuring overall lateral knee stability [1,14].

### 5.2. Interaction with Other Structures

The PFL functions in concert with multiple key knee structures:

- **Cruciate ligaments (ACL and PCL):** While cruciates regulate anterior–posterior tibial translation, the PFL complements their role by resisting external rotation and lateral translation, especially in knee flexion, thereby reducing stress on the ACL [1,7].
- **Menisci:** The PFL contributes to joint stability by stabilizing the tibia and thus protecting the menisci from excessive stresses that could cause damage or degeneration [7,12].
- **Popliteus muscle:** Working closely with the popliteus tendon, the PFL stabilizes the tibia during rotation, with the popliteus initiating controlled internal rotation to enable smooth knee flexion and extension [9,24]. Dysfunction in the popliteus muscle heightens the PFL’s importance in maintaining rotational stability.

### 5.3. Biomechanical Role and Studies

Biomechanical investigations underscore the PFL’s crucial contribution to posterolateral knee stability. In vitro studies have demonstrated that sectioning the PFL results in significantly increased tibial external rotation and lateral displacement, confirming its role as a restraint against varus and rotational forces [24].

The PFL acts synergistically with the FCL and popliteus tendon to distribute mechanical loads and prevent abnormal knee motions, particularly during high-stress activities such as jumping and cutting [23].

In vivo imaging studies using MRI and US further confirm the PFL’s dynamic function in stabilizing the knee during movement, with structural alterations such as thinning or rupture correlating with increased instability in posterolateral corner injuries [25,26].

Moreover, injury to the PFL severely compromises the knee’s ability to resist external rotation and lateral translation, reinforcing the need for its preservation or reconstruction during surgical management of complex PLC injuries [27].

Biomechanical insights have directly influenced surgical approaches, promoting anatomically based reconstructions that restore PFL function and reduce post-operative instability [24].

## 6. Pathology Associated with the Popliteofibular Ligament

The PFL is essential for maintaining knee stability, especially within the PLC. Injuries or degenerative changes to the PFL can lead to significant knee instability, impairing dynamic functions such as pivoting, cutting, and sudden directional changes. These pathologies frequently occur in high-impact sports and are often accompanied by damage to other ligamentous or meniscal structures [1,27].

### 6.1. Injuries and Trauma

The PFL is susceptible to various traumatic injuries, especially during activities involving high rotational forces or varus stress. Sports such as football, basketball, and skiing pose significant risk due to rapid changes in direction and pivoting movements [14].

#### Mechanisms of Injury

- Excessive varus force with external tibial rotation causes overstretching or tearing of the PFL, often combined with injury to the FCL and PT [1];
- Direct trauma or high-energy impacts, including falls or car accidents, may lead to severe PFL disruption and posterolateral instability [28].

**Clinical Presentation**

- Patients typically report lateral knee pain, instability during rotational movements, and feelings of the knee “giving way” [29,30];
- Physical examination may reveal varus laxity and positive dial or posterolateral drawer tests, indicating PLC injury [27].

**Diagnostic Challenges**

- PFL tears are frequently missed due to their co-occurrence with other ligamentous injuries and nonspecific symptoms [29,30];
- Variants such as bifurcated or double-band PFL may mask ligament damage until instability progresses [1].

### 6.2. Degenerative Changes

Chronic stress and aging can induce degenerative alterations in the PFL, impacting its mechanical integrity and contributing to progressive knee instability [14].

#### 6.2.1. Pathophysiology

- Repeated microtrauma leads to fiber disruption, decreased elasticity, and collagen density reduction [28];
- Chronic inflammation may result in fibrosis, scar formation, and ligament thickening or thinning [29,30].

#### 6.2.2. Clinical Impact

- Degeneration reduces the ligament’s ability to stabilize the knee, increasing risk for secondary injuries and osteoarthritis development [14,31];
- Patients may experience chronic lateral knee pain, swelling, and functional decline.

### 6.3. Diagnosis

Accurate diagnosis of PFL pathology requires integration of clinical tests and imaging modalities.

#### 6.3.1. Clinical Tests

- The Dial Test assesses increased tibial external rotation at 30° and 90° flexion, indicating posterolateral instability [27];
- The Posterolateral Drawer and Varus Stress Tests evaluate tibial translation under applied stresses [1].

#### 6.3.2. Imaging

- MRI is the gold standard for visualizing PFL tears and associated PLC injuries, with 3T MRI offering superior resolution [25,32];
- US allows dynamic assessment and is valuable for detecting partial tears or degenerative changes [26].

#### 6.3.3. Combination Approach

- Using multiple diagnostic modalities improves sensitivity and specificity in detecting complex injuries [24].

### 6.4. Advanced Imaging Modalities and Visualization of PFL Variants

Recent advances in imaging have enhanced the visualization of PFL morphology and pathology.

- **3D MRI reconstructions** provide detailed spatial anatomy and aid preoperative planning [33];
- **AI-assisted segmentation** automates ligament identification and classification, potentially standardizing assessments [34];
- **High-frequency US** offers real-time, dynamic evaluation especially useful when MRI is contraindicated [35].

The comparison of imaging features for different PFL variants is summarized in Table 2.

### 6.5. Treatment and Management

Management of PFL injuries requires an individualized approach depending on the extent of damage, anatomical variant, and degree of knee instability. Based on the classification proposed by Olewnik et al. [7] (see Section 4.1, Table 1, Figure 1 and Figure 2), treatment is divided into conservative and surgical modalities.

#### 6.5.1. Conservative (Non-Surgical) Treatment

Conservative management is primarily indicated for patients with partial PFL tears, especially those with Type I variants where mechanical and dynamic knee stability is preserved [28]. It is also appropriate for patients contraindicated for surgery or those opting for rehabilitation-based therapy.

The main goals are the restoration of muscular strength and neuromuscular control, focusing on the lateral musculature supporting knee stability such as the quadriceps, sartorius, and gluteal muscles [28,31]. Rehabilitation programs typically include the following:

- Proprioceptive and neuromuscular exercises to improve joint position sense and dynamic stability, reducing reinjury risk [27];
- Gradual progression of range of motion and strengthening while avoiding excessive rotational or varus stress in early healing [29];
- Use of lateral knee braces or orthoses to provide mechanical support during the acute and early recovery phases [31].

Treatment should be personalized considering ligament injury severity, concomitant damage, and patient activity level. Athletes often require intensive, sport-specific rehab focused on dynamic stability to ensure safe return to activity [27,31]. Monitoring includes regular clinical stability tests (e.g., Dial Test, varus stress test) and imaging follow-up via ultrasound and high-resolution 3T MRI to assess ligament healing [24,26].

Conservative treatment may be insufficient for extensive tears, mechanical instability, or high-demand patients, warranting surgical intervention [29,30,31,36].

#### 6.5.2. Surgical Treatment

Surgical reconstruction is indicated in complete or complex PFL injuries that cause significant posterolateral knee instability, especially in anatomical Types II and III variants, where structural complexity necessitates precise restoration of multiple ligamentous bands [31,36]. Surgery is also considered after failed conservative treatment or in multi-ligamentous PLC injuries involving other stabilizers such as the fibular collateral ligament or PT [28]. Early reconstruction is crucial to prevent secondary cartilage damage and post-traumatic osteoarthritis [10].

##### Surgical Techniques Include the Following

- **Single-Bundle Reconstruction:** Appropriate for Type I variants characterized by a single robust ligament. This involves creating a single fibular tunnel for graft passage, typically with autologous gracilis or semitendinosus tendons, emphasizing precise tunnel placement and graft tensioning to restore native biomechanics and avoid overconstraint or laxity [13,24].
- **Multi-Bundle Reconstruction:** Required for Types II (bifurcated ligament) and III (double ligament) variants, involving multiple tunnels or fixation points to anatomically replicate both bands and restore physiological stability and rotational control [20]. Both open and arthroscopic techniques are used; arthroscopy offers minimal invasiveness and enhanced visualization but demands advanced surgical skill and thorough preoperative imaging [31].
- **Graft Selection:** Autografts are preferred for superior incorporation, reduced immunogenicity, and long-term outcomes [36]. Allografts reduce donor site morbidity and operative time, especially in multi-ligament or revision surgeries [8]. Choice depends on patient factors, graft availability, and surgical goals.
- **Technological Advancements**: Use of 3D imaging, computer-assisted navigation, and 3D-printed patient-specific anatomical models improves surgical planning and precision, particularly in complex anatomy cases [20,33].

##### Postoperative Rehabilitation (Concise)

This is structured in phases of immobilization, early mobilization, strengthening, and functional training. The program is individualized based on the extent of reconstruction and anatomical variant to balance graft protection with restoration of knee stability [31].

##### Outcomes and Complications

Surgical reconstruction generally results in high rates of restored knee stability and functional recovery, with most patients returning to pre-injury activity levels [8,36]. Common complications include graft failure, tunnel malposition, neurovascular injury, and persistent posterolateral instability [21,22]. These are often linked to inaccurate preoperative diagnosis, technical errors, or the incomplete restoration of complex variants. Although revision surgeries are feasible, they tend to have poorer outcomes due to scarring and altered anatomy, highlighting the importance of early, precise primary treatment [31].

## 7. Clinical Variability and Its Importance

The PFL shows significant clinical and morphological variability that impacts knee stability, injury risk, diagnosis, treatment, and rehabilitation. Variations in size, shape, attachment points, and presence of accessory bands can alter knee joint stability and influence approaches to reconstruction and rehabilitation. Understanding this variability is crucial for tailoring individualized treatment and optimizing patient outcomes.

### 7.1. Treatment and Rehabilitation Implications

Morphological variants of the PFL (Types I–III) guide treatment strategies. Patients with Type I (single robust ligament) often respond well to conservative rehabilitation focusing on strengthening lateral stabilizers, proprioception, and restoring range of motion [28]. Types II and III, due to complex or double-band structures, may require more intensive rehab or surgical intervention, especially when high-stress activities or instability occur [27,31].

Surgical repair or reconstruction is indicated for complete ruptures or significant damage, particularly in complex anatomical variants. Postoperative rehabilitation emphasizes multiphase protocols beginning with early mobilization, progressing to strength and neuromuscular control exercises, and concluding with sport-specific functional training [31]. Type III variants generally require longer rehabilitation due to complexity and risk of compensatory dysfunction [27].

### 7.2. Role in Sports Medicine

In athletes, PFL variability critically affects injury diagnosis and rehabilitation. More complex variants correlate with higher instability and prolonged recovery [1,31]. Diagnostic imaging and clinical tests should be customized to variant anatomy to accurately assess damage. Rehabilitation protocols for athletes must emphasize dynamic stability, proprioception, and gradual progression, especially for Types II and III, to reduce reinjury risk.

Surgical planning in sports medicine requires consideration of morphological variants to select appropriate techniques, with arthroscopic reconstruction favored for complex double ligaments [24,27]. Tailoring interventions to individual anatomy ensures optimized functional restoration and safe return to sport.

### 7.3. Surgical Reconstruction Techniques and Outcomes

The surgical reconstruction of the PFL is a critical intervention for restoring posterolateral knee stability, particularly after severe injuries or chronic instability. Given the anatomical variability of the PFL (Types I, II, and III), individualized surgical strategies are essential to optimize outcomes and minimize complications.

#### 7.3.1. Surgical Methods Tailored to Anatomical Variants (Types I, II, III)

Reconstruction techniques vary according to the specific anatomical configuration of the PFL. The most common Type I variant, characterized by a single robust ligament attaching to the apex of the fibular head, can often be reconstructed using a single-fibular tunnel technique, typically employing autogenous grafts such as the gracilis or semitendinosus tendon [13,24].

For more complex anatomical types, including Type II (bifurcated ligament) and Type III (double PFL), surgical reconstruction requires meticulous planning and adaptation. These variants necessitate the creation of multiple tunnels or graft fixation points to replicate the native ligamentous anatomy accurately. Mestriner et al. [20] highlighted that dual graft routing or multi-tunnel fixation may be necessary to restore physiological biomechanics, prevent residual rotational instability, and achieve optimal graft tensioning.

Arthroscopic and all-arthroscopic techniques have gained popularity for their minimal invasiveness and enhanced visualization, permitting more precise graft placement, particularly in complex PFL variants [31]. However, these advanced methods demand high surgical expertise and thorough preoperative imaging.

#### 7.3.2. Surgical Procedure and Graft Materials

Common graft choices for PFL reconstruction include autografts (e.g., gracilis, semitendinosus), allografts, and, less frequently, synthetic grafts. Autografts remain the gold standard due to superior incorporation and reduced immunogenicity [31,36]. However, allografts provide the advantage of reduced donor-site morbidity and shorter operative times, particularly useful in multi-ligament reconstructions or revision surgeries [8].

The surgical procedure usually involves identification and preparation of the fibular head and femoral attachment sites, followed by tunnel drilling under fluoroscopic or arthroscopic guidance. Precise tunnel positioning is critical to replicate native ligament biomechanics and avoid graft impingement or failure [20,24].

In Type II and III variants, additional tunnels may be required, with grafts secured using interference screws or cortical fixation devices to ensure optimal fixation and tensioning. The choice of fixation method is influenced by bone quality, graft type, and surgeon preference [21].

#### 7.3.3. Clinical Outcomes and Complications

Outcomes following PFL reconstruction are generally favorable, with most patients achieving restored stability, improved function, and return to sports activities [8,36]. Nevertheless, outcomes are highly dependent on accurate anatomical reconstruction, graft choice, and postoperative rehabilitation.

Common complications include graft failure, tunnel malpositioning, neurovascular injury, and persistent posterolateral instability. Residual instability is often linked to incomplete restoration of complex PFL variants [21,22]. LaPrade et al. [1] emphasized the importance of addressing all injured components of the posterolateral corner to reduce the risk of recurrent instability.

Revision surgeries tend to have poorer outcomes due to scarring and altered anatomy. Early diagnosis and appropriate surgical technique are paramount to minimizing these risks [31].

#### 7.3.4. Practical Tips for Surgeons: Precise Anatomical Reconstruction and Avoidance of Pitfalls

Successful PFL reconstruction requires thorough knowledge of anatomical variants and their biomechanical implications. Surgeons should utilize detailed preoperative imaging (MRI, 3D reconstructions) to identify the PFL type and plan graft placement [25,32].

During surgery, intraoperative assessment of ligament tension and graft positioning is critical. Over-tensioning can lead to restricted knee motion, whereas under-tensioning may cause residual instability [24]. Special caution is necessary for Type III PFL, where dual-band anatomy demands careful differentiation of graft paths to replicate the native ligament vector forces [19].

Surgeons should also be aware of the relationship between the PFL and adjacent structures such as the fibular collateral ligament and PT to avoid inadvertent injury or incomplete reconstruction [1,10].

#### 7.3.5. The Role of Preoperative Planning and Imaging for Optimal Surgical Outcomes

Preoperative imaging, including high-resolution MRI and ultrasound, plays a pivotal role in surgical planning. Advanced MRI protocols (e.g., 3T, fat-suppressed sequences) and dynamic US allow for precise visualization of the PFL morphology and identification of complex variants [6,26,35].

Three-dimensional reconstructions and AI-assisted imaging facilitate preoperative mapping of attachment sites and ligament course, enhancing tunnel placement accuracy and graft orientation [33,34].

Furthermore, integration of navigation systems and intraoperative fluoroscopy can improve surgical precision and reduce complications related to graft malpositioning [24].

#### 7.3.6. Comparison of Surgical Techniques: Open vs. Arthroscopic Approaches

Surgical reconstruction of the PFL can be performed via open or arthroscopic techniques, each with distinct advantages and limitations. Open surgery provides direct visualization of the posterolateral corner anatomy, facilitating precise graft placement and management of complex anatomical variants, particularly Types II and III [31]. However, it is associated with increased soft tissue disruption and longer recovery times. Arthroscopic reconstruction, a minimally invasive alternative, offers reduced postoperative pain, shorter hospital stays, and faster rehabilitation, but it requires advanced surgical expertise and specialized equipment [31,36]. Recent developments in arthroscopic techniques aim to replicate the anatomical fidelity of open procedures while minimizing morbidity, yet concerns remain regarding the visualization of intricate PFL variants, especially bifurcated or double-banded ligaments [19]. Surgical choice depends on patient factors, surgeon experience, and specific anatomical considerations.

#### 7.3.7. Integration of Modern Technologies in PFL Reconstruction

Emerging technologies have started to transform surgical planning and execution in PFL reconstruction. Preoperative 3D printing based on high-resolution MRI or CT imaging allows the creation of patient-specific anatomical models, which enhance understanding of complex ligament variants and facilitate custom graft sizing and tunnel placement [33]. Furthermore, intraoperative navigation and robotic assistance can improve precision during tunnel drilling and graft fixation, reducing technical errors and improving the reproducibility of complex reconstructions, particularly in Type III variants with multiple insertion sites [20]. These technological advancements are still evolving, but early studies suggest improvements in surgical outcomes and reductions in postoperative complications.

#### 7.3.8. Future Directions and Innovations in PFL Reconstruction

The field of PFL reconstruction is progressing toward the integration of biological augmentation and regenerative therapies to enhance graft incorporation and long-term ligament function. Biologic scaffolds, growth factors, and stem cell therapies are under investigation for their potential to improve graft healing and reduce recovery times [31]. Additionally, synthetic graft materials with enhanced biomechanical properties are being developed to better mimic native ligament elasticity and strength, aiming to decrease graft failure rates [1]. Future research also focuses on personalized medicine approaches, incorporating genetic and molecular profiling to optimize patient-specific treatment plans. Continuous improvement in imaging modalities and biomechanical modeling promises to refine surgical techniques further, contributing to better functional outcomes and reduced rates of reinjury.

## 8. Conclusions

### 8.1. Summary of Key Points

The PFL plays a crucial role in knee joint stability, particularly within the PLC. It resists external tibial rotation and lateral translation, contributing to the maintenance of knee integrity during dynamic and high-impact movements. The PFL works synergistically with other stabilizing structures, including the FCL, the PT, and the joint capsule, to ensure optimal knee function.

Significant variability exists in the morphology of the PFL, with distinct types identified as Type I (single ligament), Type II (bifurcated ligament), and Type III (double ligament). These morphological variations have important clinical and biomechanical implications, influencing the risk of injury, diagnostic challenges, and surgical planning.

Recent advancements in imaging, such as 3D visualization and artificial intelligence (AI), enhance the ability to accurately identify PFL variants and associated injuries, supporting improved preoperative assessment and surgical precision.

### 8.2. Clinical Implications

A thorough understanding of the PFL’s anatomy, morphological variations, and biomechanical role is essential for diagnosing and managing knee instability. Morphological differences influence treatment decisions, with some cases requiring complex surgical reconstruction tailored to the specific PFL variant to restore joint stability effectively. Recognition of degenerative changes, particularly in older patients or those with a history of knee trauma, is critical to prevent chronic instability and long-term joint damage such as osteoarthritis.

## Figures and Tables

**Table 1:** PFL variability based on Olewnik et al.’s classification.

Type	Description	Prevalence
Type I	Single ligament attaching to the apex of the fibular head.	72.3%
Type II	Bifurcated ligament, with two bands attaching to different surfaces of the fibula.	8.7%
Type III	Double PFL, with one ligament originating from the popliteus tendon and the other from the musculotendinous junction.	7.3%

**Table 2:** Comparison of imaging features for popliteofibular ligament (PFL) variants.

Study	Imaging Modality	Identified PFL Type	Key Findings	Clinical Implication
Wu et al. (2024) [32]	MRI 3T	Type I, II, III	3T MRI enabled clear distinction of bifurcated vs. double-band PFLs.	Supports classification-based diagnosis and surgical planning.
Ciba et al. (2022) [35]	Ultrasound	Type I, II	US accurately identified PFL in lateral knee protocol.	Useful in outpatient evaluation; operator-dependent.
McKean et al. (2015) [23]	MRI 1.5T	Mainly Type I	Basic depiction of single-band PFL; limited resolution for complex types.	Less reliable in detecting morphological variability.
Fernandes (2016) [33]	MRI + 3D Recon	Type II, III	3D reconstructions improved visualization of complex PFL attachments.	Enhances preoperative planning and anatomical education.
Algizawy et al. (2022) [34]	MRI + AI segmentation	All types	AI-based segmentation enhanced identification and morphometric analysis.	Promotes automated, reproducible imaging-based classification.

## Data Availability

The data supporting the conclusions of this article are available within the article itself and upon reasonable request from the corresponding author.

## List of Abbreviations

PFL	Popliteofibular Ligament
PLC	Posterolateral Corner
FCL	Fibular Collateral Ligament
ACL	Anterior Cruciate Ligament
PCL	Posterior Cruciate Ligament
MRI	Magnetic Resonance Imaging
3D WE-DESS	Three-Dimensional Water-Excited Turbo Spin Echo Sequence
CTA	Computed Tomography Angiography
MRA	Magnetic Resonance Angiography
